# Beside the Seaside: Reflections on Local Green and Blue Spaces from Adults Aged over 50 in a Coastal Community

**DOI:** 10.3390/ijerph20146355

**Published:** 2023-07-13

**Authors:** Ursula Pool, Anna Kenyon, Lynn Froggett, Mark Dooris

**Affiliations:** 1 Healthy and Sustainable Settings Unit, University of Central Lancashire, Preston PR1 2HE, UK; 2 School of Medicine, University of Central Lancashire, Preston PR1 2HE, UK; 3 School of Social Work, Care and Community, University of Central Lancashire, Preston PR1 2HE, UK

**Keywords:** green space, blue space, wellbeing, coastal communities, healthy ageing, nature connectedness

## Abstract

This qualitative study examined the perceptions of potential wellbeing benefits of local green and blue spaces for adults aged over 50 years in a coastal town in North West England. At a population level, living close to green and blue spaces is associated with better mental and physical health, with the strength of the benefits being inversely related to the economic prosperity of an area. However, living in economically disadvantaged coastal communities may be associated with poorer health and wellbeing, particularly for older adults, despite the proximity to blue (and often green) space. Exploring this apparent paradox was the aim of the present study. Through semi-structured interviews with members of a community group, we gained insight into lived experiences of local green and blue spaces. The main themes we developed from analysis of the conversations were accessibility, amenities, social connections, quality of environment, and recuperation and escape. Our findings illustrate that participants valued natural spaces that were local and accessible, particularly when they felt connected to them, and were less likely to visit spaces that were further away and that were perceived as being less welcoming or accessible. This study provides insights into the experiences of adults aged over 50 living in coastal areas and suggests that policies and interventions aimed at promoting wellbeing in this demographic should consider the value of hyper-local green and blue spaces and their potential to promote connectedness to nature.

## 1. Introduction

### 1.1. Associations between Exposure to Nature and Health

The relationship between green and blue spaces (vegetation and bodies of water) and health and wellbeing has been the subject of increasing research interest in recent years, with a substantial body of evidence now showing that contact with natural environments is associated with positive health and wellbeing outcomes [1,2]. Specific health benefits associated with proximity to green spaces include improved mental health indicators such as reduced levels of stress, anxiety, and depression [3]. Additionally, access to green spaces has been associated with reduced risk of physical health problems such as cardiovascular disease [4] and respiratory illness [5]. Similarly, exposure to blue spaces has been linked to improved health outcomes, particularly in relation to mental health [6]. For example, spending time near bodies of water has been shown to reduce symptoms of anxiety and depression and improve mood [7,8]. The benefits of blue spaces may also extend to general health, with some research finding an association between self-reported good health and living close to the coast [9].

While the wellbeing benefits of green and blue spaces are well-evidenced, some natural environments may also be associated with potential risks or negative impacts on health and wellbeing, including disease vectors (e.g., mosquitos, ticks), allergens and risk of injury (e.g., from falling) and illness (e.g., from swimming in polluted water) from participating in outdoor activities. Further, natural spaces are not always regarded positively, particularly in urban areas where they may increase perceptions of danger due to fear of crime [10]. In the context of climate change, proximity to local green and blue spaces may also mean exposure to extreme weather events such as floods, which can have negative impacts on health and wellbeing [11]. However, these risks notwithstanding, exposure to natural spaces is generally beneficial for health and wellbeing [12].

The benefits of exposure to these natural environments have been demonstrated across the life course. For example, it has been shown that exposure to nature during childhood can have long-lasting benefits for physical and mental health [13,14]. Children who grow up with access to green spaces are more likely to engage in physical activity, which may help to reduce the risk of obesity and related health problems [15]. Additionally, exposure to natural environments has been linked to improved cognitive development and reduced symptoms of attention deficit hyperactivity disorder (ADHD) in children [16]. In adolescence and young adulthood, exposure to green spaces has been associated with improved mental health outcomes such as reduced levels of stress and improved mood [17]. There is also some evidence that exposure to nature during these developmental stages can have positive impacts on academic performance and social development [18]. Across adulthood, exposure to green and blue spaces has been linked to improved physical health outcomes such as reduced risk of cardiovascular disease, obesity, and diabetes [19]. Additionally, as mentioned above, spending time in natural environments has been shown to reduce symptoms of anxiety and depression and improve overall mental wellbeing. Finally, in older age, exposure to green and blue spaces has been associated with a reduced risk of dementia and stroke [20] and better general health and wellbeing [21]. Importantly for the present study, local neighbourhoods and environments have been shown to be particularly important for older adults’ health and general wellbeing, particularly in relation to the opportunities for physical activity and walking [22] (NB There is a lack of a consistent definition for ‘older adult’ in the academic literature and beyond. In this study, we refer to ‘adults aged over 50′ as a description reflecting the chronological age of our participants only: we recognise that it does not constitute or describe a homogeneous category).

### 1.2. Coastal Communities

Coastal communities are interesting because of their necessary proximity to blue, and often green, space and the associated health and wellbeing benefits [23]. However, these communities may also experience challenges that can be associated with poorer health and wellbeing outcomes. For example, coastal communities may be characterized by higher levels of social and economic disadvantage [24]. This disadvantage can manifest in a variety of ways, including limited access to healthcare, lower levels of educational attainment, and higher rates of unemployment, all of which can contribute to poorer health outcomes among individuals living in these areas, including lower healthy life expectancy. In addition to these factors, coastal communities in the UK are home to a growing number of adults beyond middle age [25], who may be particularly vulnerable to the health impacts of social and economic disadvantage. Since exposure to green and blue space disproportionately benefits people living in socioeconomically disadvantaged areas [26], it is possible that such exposure may be particularly beneficial for older adults. As the population in these areas continues to age, understanding the factors that affect and may improve the health and wellbeing of adults beyond middle age in coastal communities becomes increasingly urgent. In particular, there is a need to increase understanding of how the health and wellbeing potential of green and blue space present in coastal communities can be maximised for the benefit of the residents who might benefit from it most.

England’s coastal regions face higher than average deprivation, higher than average health inequalities, an above average proportion of adults aged over 50 and some of the worst health outcomes in England. Yet even after accounting for these differences in population structure and deprivation, health outcomes are disproportionately worse in these regions [27]. Given the substantial body of high-quality evidence demonstrating the health and wellbeing benefits of the presence and use of blue and green space, and the abundance of such spaces in coastal regions, we wanted to explore the potential for these areas’ natural resources to support health and wellbeing needs of the local population. It is possible that the health and wellbeing benefits of proximity to green and blue space could be harnessed with even more effectiveness in coastal communities, making use of the availability of these spaces, as a means to improve population health and wellbeing in such communities.

**Figure 1:**
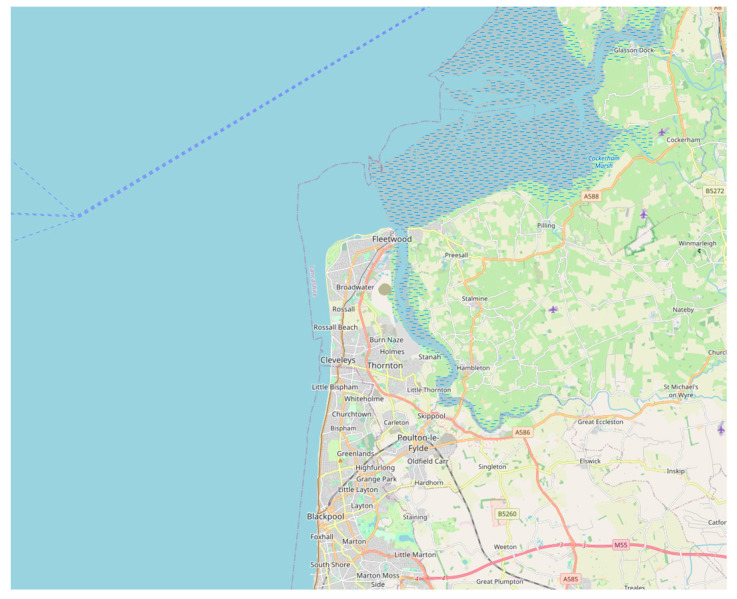
Map showing the location of Fleetwood (openstreetmap.co.uk; accessed on 4 July 2023).

Although the existence of the health and wellbeing benefits of green and blue space is well-documented, the reasons underlying these benefits are less well understood. To contribute to understanding in this area, we explored the question of what factors enable or inhibit the use of green and blue spaces in the context of healthy ageing in a particular coastal community. This study was a qualitative, open investigation, which started by asking people about their experiences of local outdoor spaces. Given the aging demographic of coastal towns in the UK and associated health problems, we focused on residents aged over 50 in our chosen location and undertook an exploration of their subjective experiences of local green and blue spaces. The location was Fleetwood, a coastal town in Lancashire, in the North West of England (see Figure 1).

## 2. Methods

### 2.1. Study Design

This study adopted a qualitative interview design, using semi-structured interviews based on a series of photographs of green and blue space in the local area. The project received ethical approval from the University of Central Lancashire’s Ethics Committee. Recruitment and data collection were undertaken together with volunteers from a social group who meet regularly in community locations in central Fleetwood.

### 2.2. Participants

Eight people volunteered to participate in the study, five female and three male. Their ages ranged from 50 to 75 years. All were attending sessions with a group, which was started to help to address loneliness and isolation in the local community. The group meets three times per week, as well as arranging other social events. Many of the people who attend do so regularly and know each other through the group.

### 2.3. Location and Demographics

Fleetwood is a coastal town in the North West of England with a population of around 26,200 people. According to the 2021 Census, the majority of Fleetwood’s population (96.9%) is White British, with a small percentage from other ethnic groups. The median age in Fleetwood is 49, and approximately 43.7% of the population is aged over 50. The percentage of people aged over 65 is slightly higher than the national average, at 22.8% (18.4% for England). The town has a higher-than-average number of retired people, with 48.8% (compared with 39.1% for England as a whole) of the population reporting that they are no longer economically active. Household deprivation is higher than average, with 64.1% of households deprived in one or more dimensions compared with 51.6% for England as a whole. The health profile of the area is below the average for England, with 40.6% of people reporting that they are in very good health (48.5% is the national average) and 9.2% of people reporting bad or very bad health (5.2% is the national average).

**Figure 2:**
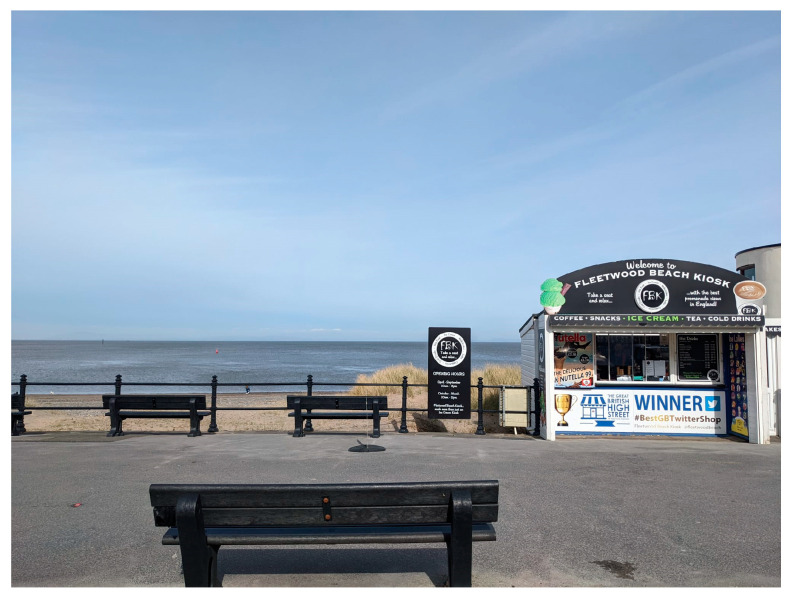
The Promenade.

**Figure 3:**
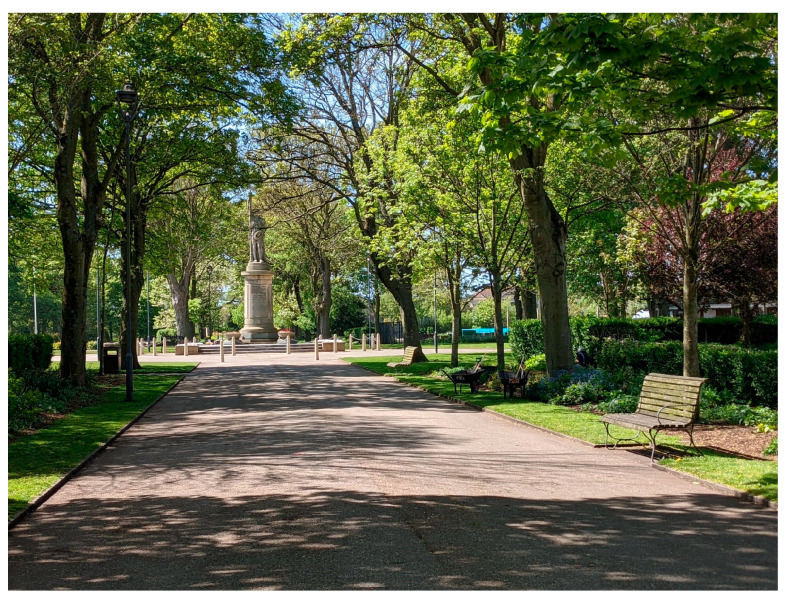
Memorial Park.

Being a coastal town, blue space is integral to the town’s identity. The town lies behind a sandy beach and shore, with dunes along some stretches. A paved promenade (see Figure 2) runs the length of the town along the sea front and beyond. There are also a number of identifiable public green spaces, including the Memorial Park (see Figure 3), Marine Hall Gardens, and the Euston Gardens.

Further out of town is Fleetwood Marshes, a wetland area. This is an important ecological site and is designated as a Site of Special Scientific Interest (SSSI) due to its diverse range of habitats and rare plant species. The marshes consist of saltmarsh, freshwater marsh, reedbeds, and ditches, which provide habitats for a wide range of bird and invertebrate species, including several that are rare or endangered. The marshes are open to the public and offer opportunities for birdwatching and nature walks. Fleetwood also benefits from an extensive network of community initiatives. In 2019, a local GP made BBC news with the ‘Healthier Fleetwood’ initiative [28] which supports links with many community groups, including the group that took part in this study.

### 2.4. Procedure

Semi-structured conversations were conducted with small groups, each consisting of two or three volunteers, during a series of visits to the regular group meetings. All the participants were already acquainted with each other from being members of the group. A table was set up away from the main group area (but still within the same large, open-plan room) far enough away for privacy in the conversation while maintaining the feeling of being part of the group. Two members of the research team took part in all the conversations. After being introduced to the research, participants were informed they would be shown a series of photographs of places containing blue and green space around Fleetwood as memory prompts, and it was explained that we were interested in their thoughts and feelings about, and experiences of visiting, these places. Locations included the beach and promenade, local parks (including both larger formal parks and smaller, less formal green spaces), and a local nature reserve. The course of each conversation was directed by the participants. Photographs were displayed on a laptop computer, with the next picture being shown once conversation about the previous picture had come to a natural end. Conversations lasted between 30 and 60 min, with the length of conversation dictated by participants and the conversation itself.

## 3. Results

Interviews were recorded digitally and transcribed, after all the conversations had been completed, by a professional transcriber. The transcripts were then analysed using reflexive thematic analysis [29,30]. This approach acknowledges the subjectivity of the process and that it is influenced by the researchers and the data themselves, as well as broader contextual factors. As detailed above, our research questions were, “How do everyday experiences of the local green and blue spaces contribute to participants’ perceptions of those spaces?” and “What factors may be important in enabling (or restricting) the use of natural spaces?”. In relation to these questions, initial themes were constructed manually by the researchers after repeated readings of the transcripts, taking into account semantic, pragmatic, and contextual factors. Themes were gradually refined and developed collaboratively through an iterative analytic process.

### 3.1. Analysis

The thematic analysis proceeded initially via an inductive approach where themes were developed from the data. First pass analysis was conducted by the two field researchers and then considered by the whole team, when emergent findings were refined by generating hypotheses followed by iterative return to the dataset. Differences in perspective were worked through until saturation was reached. In the final stage, they were set in the context of the available relevant research literature. Our goal was to build an understanding of participants’ perceptions of local natural spaces that would explain how they used them and why; similarly, which spaces they were unlikely to use and where the inhibitors might lie. The analysis was conducted within a critical realist framework [31] in which socially constructed meanings are understood in the context of participants’ experiential reality. We sequentially report, and elaborate with illustrative examples, five key themes developed through the analysis: accessibility; amenities; social connections; quality of environment; and recuperation and escape.

### 3.2. Theme 1: Accessibility

A consistent thread running through all the conversations, for all the participants, was the importance and relevance of accessibility when it came to experiences of local green and blue spaces. All except one of the participants regularly and frequently visited their local green and blue spaces, getting around them using combinations of walking, cycling, driving, and using a mobility scooter.

‘I usually drive up to there and then I walk right all the way back... I just have a nice long walk.’

‘I like to park up in the disabled [parking bay], just sit there or get my scooter out of the car. He’ll [the participant’s husband] sit and listen to music in the car and I’ll go up the prom on my scooter.’

Some participants mentioned that having an accessible route that included green or blue space affected their choice of route when not specifically visiting that space. For example, the blue space (marina area) behind the shopping centre was regarded positively with participants saying they would choose to walk alongside the water (an active choice—the shorter route passes through the shops with no view of the water). Participants said they would not make a special trip there to walk by the water, but if they were going there for the shops, they would choose to walk beside the water.

‘My favourite place we were there this morning, weren’t we? Where else can you go shopping and get the marina on the doorstep? Love it.’

‘I really like the sound of the wind, what is it that makes the noise... what are they called, the lines that they put the sails on?’

Several participants expressed sadness and a sense of nostalgia at the reduction in their own mobility, whether due to physical or practical reasons, and that being out in the green and blue spaces reminded them of this.

‘I look at people and I think, look at her, she’s walking fine, I wish I could.’

‘Since I got rid of the car we can’t go anywhere unless we travel [by public transport].’

‘Why I like going to there is because it gets me out and about and it’s nice. You can see the kids playing, you see people perhaps sat on the sand and it reminds me of when I used to be able to get on the sand. I can’t go on the sand now.’

The Marshes nature reserve is further out of town and was perceived as being less accessible to get to, despite being on a bus and cycle route and having parking facilities. It was also seen as being awkward to get around in the reserve, despite it having wheelchair-accessible walkways. However, there was a sense of risk and potential danger attached to the Marshes which exceeded issues of physical accessibility and was far less pronounced with any of the other local natural spaces

‘What if the wheels slip off the side?’

‘There is a big tick problem there at the moment, so I’m keeping away.’

Our interpretation of the data was that it was not only objective accessibility that mattered (being accessible for people in general) but also subjective accessibility (the question of whether it was possible for them in particular), and whether the barriers were as psychological as they were physical. Although one of the participants expressed positive views about the Marshes, most participants reacted more negatively and viewed this area as a place for other people, not themselves.

‘Well, I suppose they leave it like that as a as a natural place for people to go and watch for the wildlife and the birds to come. Yeah. So, like some people might enjoy going. Some people might like to get away.’

Amenities were seen as important to the accessibility of a location. For example, when describing what they did when visiting local green and blue spaces, most participants mentioned the importance of a place to sit, with some saying they would not visit unless there was a bench to sit on.

‘I wouldn’t go because I wouldn’t want to stand … I’m not one for standing. I like to sit down.’

Sitting to look at and enjoy the view was important for some participants.

‘It’s a lovely place to sit on the bench and look out over the water.’

Participants also used the benches to sit and eat or drink, usually for snack consumption or drinks, with tea being mentioned the most, followed by ice cream. Meals were also mentioned, including packed lunches and takeaways. One participant mentioned coming to sit on the bench in the Pocket Park because it was possible to connect to the Wi-Fi from the adjacent public library building, highlighting the combined appeal of both amenities.

‘It’s close to the library and you used to be able to use their Wi-Fi. It was close enough to the library to use the Wi-Fi, that’s why I used to sit there.’

Accessibility combined with sitting opportunities was appreciated. When talking about the Pocket Park, one participant said: ‘It’s just there [i.e., right in town, next to the library]. It’s lovely. Because, yeah, have a cup of tea and a cake and just sit down and think about things.’

This idea of green spaces inviting quiet contemplation and ‘just sitting’ came up several times, with participants appreciating the value of simply being in the space, as opposed to taking part in any particular activity.

‘I’ve sat on them benches; I was sitting on them benches on Sunday. I do like sitting on benches.’

Even for one participant who had a less positive disposition towards the local green and blue spaces, a comment about a photo showing the marina behind the shopping centre revealed that they could at least imagine themselves in the space, using it.

‘I’d sit there and get a takeaway.’

Although the participants varied in their personal mobility, the presence of amenities was appreciated by all; not only those with reduced mobility.

‘In lockdown I used to cycle from my house, which is just over two miles away from there, down to the Fleetwood beach kiosk, have a coffee, turn round and then cycle all the way home. And it was fantastic because you’ve got all this beautiful scenery, the fresh air, with refreshment at the end, so it was marvelous.’

In this case, the amenity of the coffee kiosk and the off-road cycle route was important to the participant.

### 3.3. Theme 2: Social Connections

Participants often mentioned experiencing their local green and blue spaces with other people—most commonly family, particularly children and grandchildren. There was also a sense of appreciation that being in these spaces created a special kind of quality time with family.

‘We go [for] a walk and my son hasn’t been very well, so we’ve been going out walking with him. We’ve got a little bench that we sit at the back, see like that bench up at the back there, we would sit up there and have a chat and just relax for an hour. And sometimes, it’s amazing how you chat, you know, if you’re in the house you maybe don’t, but if it’s just him and me, we sit on the bench, it’s amazing how you just chat away. It brings out, it brings different subjects up, you know.’

Although some participants highlighted enjoying these spaces alone, the meaning attached to visits with family seemed to be particularly important, including remembering visits when they were children themselves, evoking a sense of happy nostalgia.

‘I was always up on the beach as a child… In the holidays we were up there nearly every day.’

Several participants mentioned that local schoolchildren planted the boxes in the Pocket Park, and that was an aspect they appreciated about it. It made them feel ‘lifted.’ It also demonstrated a feeling of community, and there was an assumption that the children themselves benefitted from taking part in the planting activity.

‘You feel quite proud, even though it might not be you who’s done it. You feel proud that the people who have done it should be the children.’

‘I think it’s really good and then inspires young children when they maybe never planted a thing in their life, you know.’

### 3.4. Theme 3: Quality of Environment

Sensory features of the environment were mentioned often, and treated as being important, for example, ‘fresh air’; views (both expansive and smaller scale); noise (pleasant noises such as wind, and unpleasant noise, such as from traffic); colours of flowers and ‘greenery’. Participants appreciated and valued these qualities, which were important in their perceptions of and motivations for visiting the spaces. One participant liked the Memorial Park because of its tranquility as well as the accessibility.

‘It’s just nice and calm and quiet… no cars… and it’s near my home.’

Similarly, the Pocket Park was seen as a small area of tranquility on a busy street of the town.

‘It’s beautiful. It’s a little haven of quiet.’

In general participants preferred the park to the beach/promenade.

‘You don’t hear much other than the nature. But when you’re up at the beach, you see other people talking and all that. But when you’re in there it’s a lot more quiet with nature.’

However, there were still many positive comments about the quality of the blue space, including the beach.

‘Just look at that photo, how lucky are we to have that on our doorstep.’

There was positivity about the even smaller pockets of green space, such as street planters containing flowers.

‘It makes you feel as if people care about the environment. Planting and things.’

‘It makes you think I should remember maybe to dress with a bit more colour myself.’

### 3.5. Theme 4: Recuperation and Escape

Participants spontaneously (without prompting from the researchers) talked about the wellbeing benefits they perceived from the local green and blue spaces, including the positive feelings they generated, and the sense of psychological escape.

‘That is a nice park, I go there a lot… [it makes me feel] quite calm. When you’re sitting on a bench… that is mindfulness, for me, because I find it very, very difficult to relax. That’s why I come here.’

Several participants individually mentioned the idea of holidays and being away, and linked these places with those feelings, suggesting that being in these spaces could provide a certain psychological escape or respite from everyday reality. This seemed particularly apparent with the blue spaces, as opposed to the photos only containing green space.

‘It’s just like you’re there at the seaside.’

‘It makes you feel as if it’s the Riviera… in France… every time I’m there… it feels as if you’re abroad.’

‘Think about Benidorm—we didn’t need to when I was a kid. [because the local beach was just as good]’

‘Well, the sky’s really nice and then if you look out, you know, you just see that edge of the water. You don’t know what’s over it, you just see the edge and the sand. You could be anywhere in the world really, you could be, it’s very nice.’

It was notable that most participants seemed not only to derive benefit from their experiences of local green and blue space, but were aware of those benefits, sought them out deliberately and appreciated them.

‘There’s no point living by the seaside otherwise, is there?’

‘Apparently, we have the freshest air in Britain.’

‘I think it’s really important for your mental health that you get outside into the greener areas.’

‘You can’t underestimate the healing power of nature, whether you realise it or not. Being sort of amongst trees and hearing them rustle in the breeze, it’s good for your mental health.’

These results provide insight into how the participants perceived local natural spaces, through the themes we developed from the data. Accessibility, both in terms of practical access and personal perception, was seen as essential, with amenities, especially seating options, playing a key role. Natural spaces also fostered social connections and promoted community engagement, with shared activities contributing to a sense of pride and social bonding. The quality of the environment, particularly tranquility and sensory elements, is reflected in the participants’ preferences for quieter, nature-filled spaces, but these spaces did not necessarily need to be wild. Lastly, participants perceived these spaces as offering recuperative benefits, providing feelings of calm, mindfulness, and a psychological retreat.

## 4. Discussion

This analysis aimed to explore the ways in which Fleetwood residents aged over 50 perceived their local green and blue spaces. We hoped that gaining some insight into this would help in the understanding of the factors that are important in harnessing the health and wellbeing benefits of green and blue spaces in socioeconomically disadvantaged coastal communities, particularly for the ageing population. We also hoped to identify both the practical and psychosocial enablers and inhibitors in the use of local natural space, as it is these that have implications for planning and for public health.

We reported four overarching themes which provided insight into the participants’ experiences and perceptions: accessibility; amenities; social connections; quality of environment; and recuperation and escape. Taken as a whole, the analysis suggests that there were two key factors when it comes to explaining why a particular green or blue space would both be used/visited and provide a boost to wellbeing. Firstly, the practicalities were important. It was essential that a space was accessible—both in terms of reaching it, and in terms of moving around within it, and being able to do that smoothly, safely, and without fear. Part of this involved being able to negotiate the space on one’s own terms at one’s own pace, and the importance of provision of benches and other facilities to enable this proved to be significant for many participants. This finding is in line with previous studies that have found facilities, such as benches and cafes, to be important in promoting and motivating the use of parks among adults classified as being ‘older’ [32,33]. The availability of benches and toilets have been found to be especially important since they can potentially facilitate the ability to walk for more extended periods [34,35].

While practical facilities were important, our analysis suggests that it was not only physical accessibility and enabling of mobility that mattered. A further finding was the importance of a space being not only objectively, but also psychologically/subjectively, accessible and safe. That is, participants needed to identify with the space and feel that it was for them, particularly in terms of feeling comfortable there psychologically. This identification was easier to achieve when it came to the local spaces that were encountered more frequently and easily during the course of everyday life. This is broadly in line with earlier research that found that factors such as safety, accessibility, and personal perception could affect older adults’ relationships with green and blue space [36]. The presence of a bench, for example, was not merely a convenience but gave the occupants an opportunity to appropriate the space for themselves for everyday purposes like snacking and picnicking, or quiet contemplation. Essentially it ‘domesticated’ the space, rendering it less alien and hence less fraught with risk. A key finding of the study is that unmediated contact with wildness can be daunting or even frightening for some. Access to blue or green spaces is not merely a function of the fact that they are there and easy to get to, but rather a function of the fact that people make use of them, integrating them into routines and leisure activities. The perception that the spaces that were further out of town were more likely to be dangerous or unsuitable in some way was a deterrent to visiting. Even for the participants who did not have any particular limitations on their mobility, accessibility (e.g., bike paths) and being close to town were important for their frequency of access and use.

The other key factor suggested by our analysis is the idea that a personal connection with the space, or a personal meaning linked with it, was important. That is, although the space needed to be accessible and pleasant to be in, having meaningful personal connections also seemed to matter. For example, knowing that the Pocket Park had been planted by local children gave this space extra meaning and positive associations, which created a particular fondness for, and enjoyment from, experiencing it. For other spaces such as the beach and the Memorial Park, participants linked their experiences with family visits which imbued the spaces with significance and positive associations. This finding is compatible with a strand of research around ‘nature connectedness’, which focuses on the emotional and psychological bonds that people have with nature [37] and the links between nature connection and wellbeing [38]. This work is consistent with the large body of research demonstrating the restorative and replenishing properties of natural spaces that can positively impact on feelings of wellbeing [39,40]. Further, our findings support the idea that the personal and social meaning attached to certain natural spaces may underlie some of their wellbeing benefits. It was interesting that for the participants in this study, the spaces with meaning, towards which they demonstrated a sense of connectedness, were often the smaller, local spaces, which they would be likely to encounter during the course of everyday life. The links to family memories or thinking of the children who planted the flowers, for example, gave these hyper-local spaces significant extra meaning and value. The further away spaces did not have those connections and were not valued, used, or experienced in the same way by our participants. Whether it might be possible to create such connections for the spaces located further away is an open question but our findings suggest that that the provision of physical infrastructure such as walkways is not in itself sufficient. Enabling access to and enjoyment of such spaces needs to be strategic and sensitively attuned to what makes nature socially meaningful and therefore less alien and anxiety-provoking.

In this study, we looked at green and blue space together, as a representation of the natural environment. However, it is not necessarily the case that green and blue space have an identical relationship with wellbeing [41]. Although the main themes we constructed applied to all the natural spaces our participants talked about, there were some subtle differences in emphasis between the responses to green and blue space. While both were clearly seen as restorative, the settings that were predominantly green, such as the parks, evoked feelings of peacefulness and calm; however, the blue space settings, such as the beach and the marina, were more likely to bring about talk of escape and holidays. Our investigation was not looking for differences in perceptions of green and blue space, but these observations suggest that future research into possible differences and different opportunities presented by these different natural settings might be warranted, particularly in coastal locations where green and blue space tend to both be present, and could potentially have effects unique to such locations.

The study adds insights to the discourse on natural spaces by focusing on the experiences and perceptions of people aged over 50. It illustrates the multidimensional nature of accessibility, emphasizing that this is not only about physical access but also encompasses elements of personal perception and comfort. In addition, the study demonstrates the role of natural spaces in fostering social connections and promoting community engagement, thus enriching our understanding of the social function of these spaces beyond their aesthetic and recreational roles. Our results indicate the importance of sensory aspects of the environment, providing support for a more holistic approach to understanding and designing natural spaces. The findings reinforce the body of evidence supporting the restorative benefits of natural spaces, and show that older individuals are not only passive beneficiaries but also active seekers who understand some of these benefits.

Bearing in mind the limitations of this study—our participant sample was small and not representative of the population of Fleetwood, including those aged over 50—it is nonetheless possible to make recommendations stemming from our findings for policy makers who would like to make urban green and blue space more available for more people. These include:

- Provide many hyper-local green and blue spaces, even if small;
- Maximise quality of the sensory characteristics of these urban natural spaces;
- Ensure natural spaces have practical amenities, especially seating;
- Make these spaces accessible for residents going about their everyday lives, without having to make a special journey, including people with restricted mobility;
- Involve the local community in the creation and upkeep of natural spaces, thus imbuing them with community meaning and significance that many residents will be able to identify with.

## 5. Conclusions

The participants in this study valued the natural spaces which they felt were the most local and accessible to them, particularly when they felt connected with those spaces. Prioritising access to smaller, local areas of high-quality green and blue space may be important for harnessing the wellbeing benefits for residents aged over 50. Encouraging the use of natural spaces by making these welcoming to local population groups could provide a mechanism to reduce health inequalities by maximising the health-supporting opportunities that such spaces offer. However, creating spaces which have meaning for local people, for example, by involving them or their families and friends in the development of those spaces, may also be important. Future research might focus on the best ways of creating meaning and value for people in their (current and future) local green and blue spaces.
